# Trypsin Induced Degradation of Amyloid Fibrils

**DOI:** 10.3390/ijms22094828

**Published:** 2021-05-02

**Authors:** Olga V. Stepanenko, Maksim I. Sulatsky, Ekaterina V. Mikhailova, Olesya V. Stepanenko, Irina M. Kuznetsova, Konstantin K. Turoverov, Anna I. Sulatskaya

**Affiliations:** 1 Laboratory of Structural Dynamics, Stability and Folding of Proteins, Institute of Cytology, Russian Academy of Sciences, 4 Tikhoretsky Avenue, 194064 St. Petersburg, Russia; sov@incras.ru (O.V.S.); 4evamkh@gmail.com (E.V.M.); lvs@incras.ru (O.V.S.); imk@incras.ru (I.M.K.); ansul@mail.ru (A.I.S.); 2 Laboratory of Cell Morphology, Institute of Cytology, Russian Academy of Sciences, 4 Tikhoretsky Avenue, 194064 St. Petersburg, Russia; m_sulatsky@mail.ru; 3 Institute of Physics, Nanotechnology and Telecommunications, Peter the Great St. Petersburg Polytechnic University, Polytechnicheskaya 29, 195251 St. Petersburg, Russia

**Keywords:** amyloid fibrils, proteolytic degradation, trypsin, stability, fragmentation, cytotoxicity, florescent probes, thioflavin T (ThT), 1-anilinonaphthalene-8-sulphonate (ANS)

## Abstract

Proteolytic enzymes are known to be involved in the formation and degradation of various monomeric proteins, but the effect of proteases on the ordered protein aggregates, amyloid fibrils, which are considered to be extremely stable, remains poorly understood. In this work we study resistance to proteolytic degradation of lysozyme amyloid fibrils with two different types of morphology and beta-2-microglobulun amyloids. We showed that the proteolytic enzyme of the pancreas, trypsin, induced degradation of amyloid fibrils, and the mechanism of this process was qualitatively the same for all investigated amyloids. At the same time, we found a dependence of efficiency and rate of fibril degradation on the structure of the amyloid-forming protein as well as on the morphology and clustering of amyloid fibrils. It was assumed that the discovered relationship between fibrils structure and the efficiency of their degradation by trypsin can become the basis of a new express method for the analysis of amyloids polymorphism. Unexpectedly lower resistance of both types of lysozyme amyloids to trypsin exposure compared to the native monomeric protein (which is not susceptible to hydrolysis) was attributed to the higher availability of cleavage sites in studied fibrils. Another intriguing result of the work is that the cytotoxicity of amyloids treated with trypsin was not only failing to decline, but even increasing in the case of beta-2-microglobulin fibrils.

## 1. Introduction

The development of a number of serious diseases, so-called conformational diseases, which include Alzheimer’s, Parkinson’s, prion diseases, etc. [1,2,3,4,5,6,7], is associated with the accumulation of amyloid fibrils in various organs and tissues of the human body. The appearance of an amyloidogenic protein in the body may be due to the proteolytic cleavage of its precursor, which itself does not tend to aggregate to form amyloid fibrils [8,9,10,11]. For example, β-secretase generates several short peptides from the amyloid precursor protein (APP). The formation of amyloid fibrils from one of these peptides (42 amino acid residues long) is associated with Alzheimer’s disease [8]. Thus, the processing of precursor proteins with the participation of proteolytic enzymes leads to the formation of native proteins and peptides [12], including those with amyloidogenic properties.

In addition, proteases can modify native proteins and induce, accelerate, slow down, or even inhibit their aggregation [13,14,15]. In particular, it was shown that the rate of whey protein concentrate (WPC) aggregation increases significantly upon treatment with trypsin and noticeably decreases upon treatment with protease A, pepsin, and protease M [13]. This indicates the possible involvement of proteolytic enzymes in the fibrillogenesis of various proteins. Recent studies also indicate that proteases, in particular trypsin, NET-associated elastase, proteinase K, and HTRA1, can lead to degradation of mature amyloid fibrils, including those accumulating in amyloid plaques in vivo [16,17,18,19,20,21]. This is probably due to the main function of proteolytic enzymes, which consists of the degradation of misfolded proteins [22,23,24,25,26,27]. Assessment of fibril resistance to proteolysis is currently used as an effective approach to the analysis of external factors influence on the structure and stability of amyloids [18]. However, despite the widespread use for the study of amyloids, the mechanisms of fibril degradation under the influence of proteases and changes in the structure and cytotoxicity of amyloids, induced by the treatment with these enzymes, remain poorly understood.

In this work, we investigated the process of degradation of amyloid fibrils formed from two amyloidogenic proteins with significantly different primary, secondary, and tertiary native structure after the action of the trypsin serine protease, which is widely used to estimate the resistance of fibrils to proteolysis. Trypsin is one of the main digestive enzymes and effectively catalyzes the hydrolytic cleavage of proteins and peptides at the carboxyl side of L-lysine or L-arginine residues. We revealed changes in the structure and stability of amyloid fibrils after the action of trypsin using a number of spectroscopic approaches, including fluorescence of the amyloid-specific dye thioflavin T (ThT) and the hydrophobic probe 1-anilinonaphthalene-8-sulfonic acid (ANS), as well as transmission electron microscopy (TEM) for amyloid fibrils imaging. The cytotoxicity of amyloid fibrils before and after trypsin exposure was evaluated using the *HeLa* cell line. Lysozyme and beta-2-microglobulin were chosen as amyloidogenic proteins in this study. Accumulation of amyloid fibrils of these proteins leads to the development of hereditary lysozyme systemic amyloidosis (ALys amyloidosis, [28]) and dialysis-related amyloidosis (DRA, [29]), respectively. Fibrillogenesis of lysozyme was induced using two alternative methods, which made it possible to obtain protein aggregates with a polymorphic structure [30]. Thus, experiments were carried out using the amyloid fibrils with different types of morphology, as well as with different primary and secondary structures of amyloid-forming proteins, which allowed us to draw a conclusion about the universality of the observed degradation effect of trypsin.

## 2. Results

### 2.1. Mechanism of Amyloid Fibril Degradation Induced by Trypsin: Fragmentation, Disordering, and Monomerization

Model lysozyme amyloid fibrils, the accumulation of which leads to the development of hereditary lysozyme systemic amyloidosis (ALys amyloidosis, [28]), were selected as one of the objects of study. Literature data indicate that trypsin exhibits proteolytic activity in a narrow range of neutral pH [31,32,33]. In this regard, lysozyme amyloid fibrils prepared using the standard protocol (see Materials and Methods) were transferred to a solution with pH 7.4 to analyze the action of trypsin. Previously, we showed that mature lysozyme fibrils, transferred to physiological conditions, were stable for at least a week [34]. Based on the literature, the ratio of trypsin and amyloid-forming protein in the experiment was chosen equal to 1: 125 [35,36,37].

Considering the fact that trypsin is susceptible to autolysis, first of all, we checked the rate of this process in phosphate buffer with pH 7.4 at 37 °C using fluorescence spectroscopy and SDS-PAGE (Figure S1). This allowed us to determine the duration of the experiment on fibril degradation. It turned out that, under the chosen conditions, trypsin actually underwent autolysis, and after 24 h, the band corresponding to this protein on SDS-PAGE became indistinguishable (Figure S1A). Similar results, indicating the autolysis of trypsin during the day, were obtained when analyzing changes in the intrinsic tryptophan fluorescence intensity and parameter *A* of the protease (parameter *A* is the ratio of fluorescence intensities at wavelengths of 320 and 365 nm, sensitive to the position and shape of the fluorescence spectrum) [38,39]. Thus, it was concluded that, under the chosen conditions, trypsin could be active for at least 24 h. For example, residual activity of trypsin was observed in a concentrated solution of BSA even after prolonged incubation (Figure S2). Since the weight ratio of trypsin to amyloidogenic protein in the sample was 1 to 125, it is highly likely that trypsin predominantly affects the amyloid-forming protein, and not itself. It is important to note that trypsin was not previously incubated in neutral buffer separately but was added directly into the fibril suspension. In this regard, it was decided to study the effect of trypsin on fibrils for at least 24 h and while detectable changes are observed.

Next, we studied trypsin binding to fibrils. For this aim, amyloid fibrils after trypsin adding and the enzyme alone (control) were incubated in PBS (pH 7.4) for 10 min at 37 °C. Then, trypsin was inactivated using phenylmethylsulfonyl fluoride (PMSF), and the samples were centrifuged at high speed (18,000 rpm) for 1 h. Supernatants of both samples were collected and analyzed using SDS-PAGE under denaturing conditions (Figure S3). The results indicated the absence of trypsin in the supernatant of the sample with amyloid fibrils, although in the supernatant of the control (without fibrils), a bright band corresponding to the protease molecular weight was clearly seen (Figure S3). This means that in the sample with amyloids, all trypsin molecules remained in the pellet in a bound to fibrils state. Thus, the trypsin binding to amyloid fibrils was experimentally confirmed.

**Figure 1:**
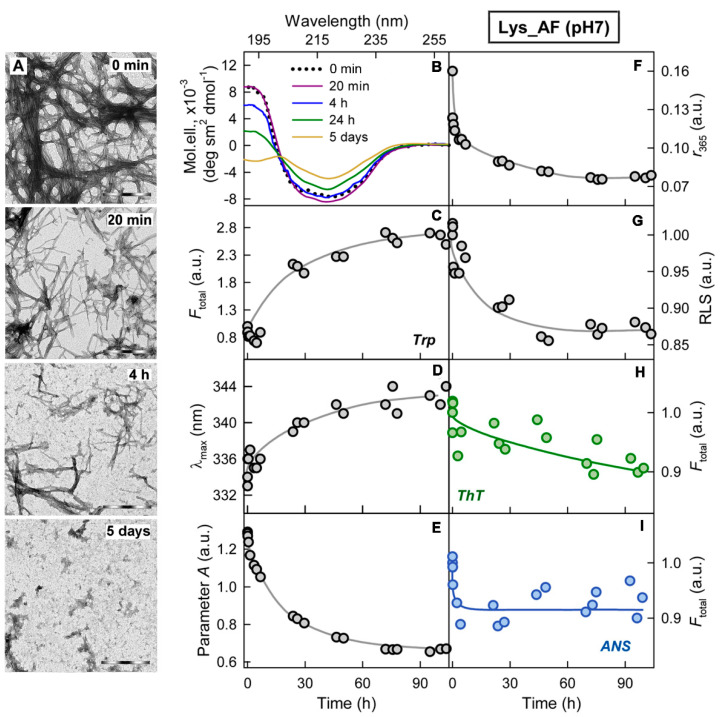
Trypsin-induced degradation of amyloid fibrils formed from lysozyme at pH 7. (**A**) The electron micrographs of amyloid fibrils in the absence and at different time points after the addition of trypsin to the samples. Scale bars are equal to 1 μm. (**B**) Changes in far UV CD spectra of amyloid samples. (**C**) Changes in total fluorescence intensity of amyloid fibrils at the excitation wavelength of 295 nm. (**D**) Changes in wavelength of the fluorescence spectrum maximum of amyloid fibrils (λ_max_). (**E**) Changes in parameter A of amyloid fibrils, λ_ex_ = 295 nm. (**F**) Changes in fluorescence anisotropy of amyloid fibrils, λ_ex_ = 295 nm, and λ_em_ = 365 nm. (**G**) Changes in Rayleigh Light Scattering of amyloid fibrils. (**H**) Changes in the total fluorescence intensity of ThT and (**I**) ANS in the presence of amyloid fibrils. The values at the panels (**C**,**H**,**I**) are normalized to the values for fibrils in the absence of the protease. The experiments were performed under physiological conditions (in 20 mM sodium phosphate buffer solution, pH 7.4) and weight ratio of fibrils to trypsin was kept at 125:1. The standard error of the mean is determined for a confidence interval of 0.95 and does not exceed 10%.

In order to study trypsin’s effect on amyloid stability, we detected changes in the intrinsic photophysical characteristics of fibrils and the characteristics of specific fluorescent probes bound to them (Figure 1). It was noted that the most significant change in the recorded characteristics occurred in the first few hours after the start of the experiment, and then smoother changes were observed over the next five days. Further, we investigated changes in the structure and properties of amyloid fibrils after them being exposed to trypsin, but we do not claim that trypsin hydrolyzes the fibrils during all five days of the experiment. These changes can both directly be caused by the enzyme present in the fibril sample (during the first day) and can be a delayed effect of short-term exposure to trypsin after its autolysis (for example, as a result of fibrils’ destabilization, which promotes their further gradual degradation).

Visualization of structural changes in amyloid fibrils by TEM at 20 min, 4 h, and 5 days after the trypsin addition was performed (Figure 1A). We observed that after the addition of the protease to amyloids suspension, defragmentation of fibrils took place; however, the formed fragments of fibrils remained ordered (for 20 min); after that, the structure of these fragments was “fluffed” (with an increase in the incubation time to 4 h). Our results indicate that the samples of fibrils five days after the addition of trypsin contain non-fibrillar aggregates of various sizes (from several tens of monomeric subunits to the size of a mature amyloid fibril). These aggregates can be a result of fibril degradation induced by trypsin, as well as association of the products of proteolytic degradation of fibrils with each other. It should be noted that along with the products of proteolytic degradation full-length mature amyloid fibrils were retained in the sample.

We also checked whether degradation of amyloids induced by some external proteolytic activity (not coming from the trypsin) occurs in the control sample with no trypsin added. Tested samples were incubated at 37 °C in phosphate buffer. Intact amyloids were stable during at least a one-week period, as confirmed by TEM (Figure S4).

**Figure 2:**
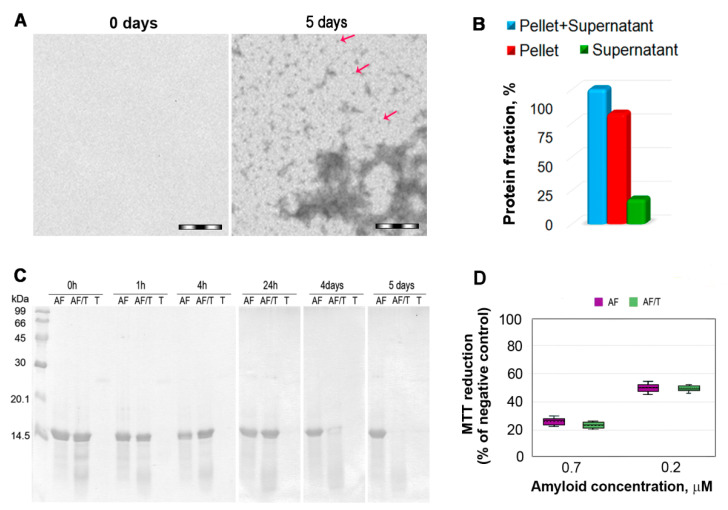
Stability and inhibitory effect on cells of exposed to trypsin amyloid fibrils formed from lysozyme at pH 7. (**A**) The electron micrographs of supernatant collected by centrifugation of amyloid fibrils before and five days after the addition of trypsin. Scale bars are equal to 200 nm. The estimated size of aggregates corresponds to 20 (shown by red arrows) or more monomeric protein molecules as evaluated by ImageJ. (**B**) Redistribution of the protein between supernatant and pellet obtained by centrifugation of amyloid fibrils treated with trypsin. (**C**) Trypsin-induced degradation of lysozyme amyloid fibrils detected by SDS-PAGE under denaturing conditions. The amyloid fibrils alone (AF), in the presence of trypsin (AF/T) and trypsin (T) solutions, were incubated in PBS at 37 °C, and aliquots were taken at different times. Molecular weight of lysozyme and trypsin is 14.3 and 23.3 kDa, respectively. (**D**) Dose-dependent inhibition effect of amyloid fibrils on HeLa cells determined by MTT assay. Cells were exposed to amyloid fibrils, intact (AF) or five days after the addition of trypsin to the sample (weight ratio of fibrils to trypsin was kept at 7.5/1) (AF/T), at indicated concentrations for 24 h. Data are expressed as medians and interquartile ranges, whiskers denote 1.5 × IQR. Experiments were repeated at least three times with similar results.

To assess the degree of protein aggregates degradation, samples before and five days after the addition of protease were centrifuged at high speeds (2 h, 18,000 rpm), after which the supernatant was collected and analyzed. Analysis of the supernatant sample untreated with protease showed the absence of fibril fragments and disordered aggregates as confirmed by TEM (Figure 2A, TEM data), as well as by the closeness to 0 of its optical density. This indicates the stability of the studied aggregates and the absence of processes of their spontaneous degradation. According to TEM data (Figure 2A), degraded fibrils fragments, unstructured protein aggregates, were present in the supernatant of the sample after trypsin exposure. Using absorption spectroscopy, it was shown that the proportion of the degraded fraction in supernatant is about 20% of the initial concentration of amyloid fibrils (Figure 2B). It should be noted that degraded aggregates partially precipitated during centrifugation and large non-fibrillar associates comparable in size to amyloid fibrils were found in the pellet. Thus, the real fraction of fibril degradation products substantially exceeds 20%. We also show that increase in trypsin concentration led to an increase in the proportion of the degraded fraction in the sample (Figure S5).

The effect of trypsin on lysozyme amyloid fibrils was also analyzed by SDS-PAGE under denaturing conditions (Figure 2C). Samples of trypsin in PBS at the same concentration as in samples with fibrils, as well as amyloid fibrils in the absence of trypsin, were used as controls. Surprisingly, we did not observe a band corresponding to the molecular weight of the protease in the analyzed sample immediately after the mixing fibrils with trypsin, in contrast to the control sample (freshly prepared trypsin only) (Figure 2C, 0 h). Similar results were obtained earlier in the study of the interaction of Abeta-peptide amyloids with trypsin [19]. This indicates that the affinity of trypsin binding to fibrils is so high that this interaction does not destroy either the effect of ionic detergent (SDS) or boiling the sample (for 5 min). It should be noted that the band corresponding to trypsin did not appear in the samples with fibrils for five days. At the same time, after the addition of trypsin to the fibril suspension, the content of monomeric lysozyme first increased and then decreased (according to the analysis of the monomeric fraction ratio in the samples before and after trypsin exposure, shown in Figure S6), which confirmed the effect of protease on amyloids. We did not observe a band corresponding to the molecular weight of monomeric lysozyme five days after the addition of trypsin, which indicated an almost complete proteolytic degradation of lysozyme monomers released under the action of trypsin. In addition, a decrease in the number of protein fragments that are formed immediately after the addition of trypsin can be noted, pointing at their degradation.

Interestingly, according to the literature, compact globular lysozyme in its native state is not hydrolyzed by a number of proteolytic enzymes, including trypsin, chymotrypsin, and papain, which indicates the localization of protease cleavage binding sites within the protein globule [40]. However, these enzymes can hydrolyze denatured lysozyme [41]. We were convinced of the stability of monomeric native lysozyme exposed to trypsin using SDS-PAGE under denaturing conditions: the intensity of the band corresponding to monomeric lysozyme did not change five days after the addition of trypsin to protein (Figure S7). The fact that during this time there is a significant degradation of amyloid fibrils formed from this protein allows us to conclude that amyloid cleavage sites are more exposed than in monomeric protein.

In order to reveal the structural changes of the studied lysozyme amyloid fibrils induced by trypsin, we analyzed the results obtained using various spectroscopic methods, such as intrinsic UV fluorescence of proteins and CD spectroscopy of fibrils in the far UV region, and also an approach based on the analysis of the interaction of amyloid fibrils with fluorescent probes. The amyloid-specific dye thioflavin T (ThT) [42,43,44] and the hydrophobic probe 1-anilinonaphthalene-8-sulfonic acid (ANS) [45,46,47] were used, which are widely known probes to detect the formation of amyloid fibrils and study their structure and stability [48,49,50,51].

After the treatment of amyloid fibrils with trypsin, a significant decrease in the values of their photophysical characteristics such as parameter *A* [38,39] (Figure 1E) and fluorescence anisotropy (Figure 1F) as well as a shift in the maximum of the spectrum of intrinsic UV fluorescence of the sample to longer wavelengths was observed (Figure 1D). In addition, after trypsin exposure, an increase in the integral fluorescence intensity of tryptophan residues of amyloid fibrils was found (Figure 1C). Such changes in photophysical characteristics are usually observed during protein denaturation [49,52]. As already noted, five days after the addition of trypsin to fibrils, significant changes in the values of the recorded parameters were no longer observed (Figure 1). However, the values of these parameters did not reach the values characteristic of lysozyme in the unfolded state, in which all tryptophan amino acid residues of the protein are exposed to water, which determines the low value of the parameter *A* (*A* = 0.45) and fluorescence anisotropy (*r*_365_ = 0.05), as well as the long wavelength position of the fluorescence spectrum maximum (*λ*_max_ = 350 nm) of the sample [53,54,55,56,57]. The observed difference in the values of the photophysical characteristics of the studied fibrils, measured five days after the addition of trypsin, from the values characteristic of the protein in the unfolded state, may be due to the following facts: (1) the sample still contains a significant amount of mature intact amyloid fibrils that have not been exposed to protease and also (2) the monomers of the amyloid-forming protein in the aggregates are not completely denatured after trypsin exposure. To understand the reasons for the observed differences, we compared the samples of amyloid fibrils five days after the addition of trypsin at trypsin/protein ratios of 1:125 and 1:1. It turned out that the characteristics of the sample treated with higher concentration of trypsin were even closer to the characteristics of the denatured protein. Following that, we sedimented amyloid fibrils and large aggregates by centrifugation and evaluated the photophysical characteristics of the protein that remained in the supernatant. As we expected, these characteristics practically coincided with the characteristics of the fully denatured protein (Figure S8).

Four hours after the addition of protease to the sample with amyloids, a change in the shape of the CD spectrum of fibrils in the region of 190 nm was observed (Figure 1B). An even more significant change in the shape of the CD spectra (not only in the short-wavelength region, but also in the region of the minimum at 220 nm) was detected 24 h and five days after adding trypsin to the sample with amyloids. The obtained data indicate that the secondary structure of lysozyme subunits in the amyloid fibril changes significantly after treatment of the samples with trypsin. In order to assess the nature of these changes, we analyzed the content of various elements of the secondary structure in the sample using special software CDPro [58]. It was shown that the addition of trypsin to the studied amyloid fibrils led to an increase in the proportion of disordered structure due to a decrease in the proportion of ordered α-helical structure and β-strands in amyloid-forming proteins (Figure S9A). Considering the fact that β-strands form the backbone of the amyloid fibril, the observed changes confirm the assumptions about the degradation of amyloid fibrils. In this case, the addition of trypsin leads to a change in the structure of not only amyloidogenic (β-folded) but also non-amyloidogenic protein fragments.

Trypsin treatment of the studied amyloid fibrils was also accompanied by a decrease in the fluorescence intensity of specific dyes ThT (Figure 1H) and ANS (Figure 1I) bound to fibrils. According to the literature, ThT incorporates into the grooves formed by the side chains of amino acids of the amyloid fibril backbone along the fiber axis perpendicular to the beta-sheets [59], and ANS is a hydrophobic probe interacting with protein associates [45,46]. Thus, the dyes are likely to bind to different sites of amyloids, which is in line with the results of the work [48]. We believe that it explains the difference of kinetic dependences of fibril degradation monitored by ANS and ThT fluorescence. Different ANS and ThT binding kinetics was observed in the process of fibrillogenesis previously [60]. It can be assumed that the change in ThT fluorescence indicates degradation of amyloid fibers, and ANS fluorescence mainly reflects the fibril declasterization and degradation of non-fibrillar protein aggregates. This is in good agreement with TEM data indicating fragmentation and decreased ordering of amyloids in the presence of trypsin (Figure 1A). The significant reduction in Rayleigh light scattering (RLS) of amyloid fibril samples after the addition of trypsin confirms the disintegration of fibrillar clots and destruction of the fibrillar core (Figure 1G).

To assess the effect of trypsin on the cytotoxicity of amyloid fibrils, the metabolic activity of *Hela* cells was determined using the MTT test in the presence of amyloids before and five days after the addition of protease. First of all, trypsin at the used concentration was shown to be non-toxic to cells (Figure S10). It turned out that the level of reduced MTT was the same for samples of amyloid fibrils exposed to trypsin and for intact amyloids (data not shown). This indicates that the action of trypsin on fibrils did not change their toxicity to cells. A significant increase in the proportion of degraded fraction of amyloid fibrils in the sample with a 17-fold increase in trypsin concentration also did not lead to a change in toxicity (Figure 2D), despite a significant decrease of intact amyloids in the sample. In order to explain these results, the metabolic activity of *Hela* cells was determined in the presence of monomeric lysozyme and its fragments obtained as a result of proteolytic degradation of the protein (Figure S10) (to obtain protein fragments, protein was preliminary denatured). It turned out that monomeric lysozyme and its fragments did not affect cell viability at a concentration equal to the concentration of fibrils in the experiment. It means that if only these protein fractions were the products of degradation, the total cytotoxicity of the sample after trypsin exposure should have decreased. It can be assumed that, along with lysozyme fragments and monomers, proteolytic degradation of fibrils also results in the formation of non-fibrillar aggregates that are toxic to cells (probably even more cytotoxic than amyloid fibrils themselves). At the same time, the quantitative ratio of cytotoxic and non-cytotoxic protein fractions is such that the cytotoxic effect of non-fibrillar aggregates is leveled and, as a result, the viability of cells before and after their treatment with trypsin remains unchanged.

We tried to estimate the size of aggregates formed after trypsin exposure. We analyzed the aggregates, formed after treatment of amyloid fibrils with trypsin (trypsin/protein ratio of 1:7.5), using pseudo-native SDS-PAGE in 8% polyacrylamide gel [61]. Obtained results indicate the absence in the sample of fractions with molecular weights less than 220 kDa (Figure S11). Increase in the concentration of trypsin in the sample (trypsin/protein ratio of 1:1) led to similar results. Our rough estimate of the size of aggregates visualized by TEM (Figure 2A and Figure S12) allowed us to conclude that they consist of 20 or more monomeric protein subunits (which may explain their absence on SDS-PAGE) and have a broad size range. The linear dimensions of some aggregates are comparable to the size of intact amyloid fibrils (Figure S12). A higher cytotoxicity of such large aggregates, representing degraded amyloids, relative intact fibrils have been already demonstrated by us previously for the same fibrils treated with a protein with a chaperone activity of alpha-B-crystallin [34].

Based on the obtained results, it is concluded that trypsin-induced degradation of the studied lysozyme amyloid fibrils occurs according to the following mechanism: (1) first, amyloid clots are declustering, and amyloid fibers are fragmented; (2) then, the structure of shortened fibril fragments “decompacts” and becomes less ordered, (3) after which these aggregates degrade to monomers, which, in turn, degrade into short fragments. In this case, despite the fact that proteolytic cleavage of fibrils to short non-toxic protein fragments occurs, the total cytotoxicity of the sample after trypsin exposure does not decrease, which may indicate the presence of highly toxic non-fibrillar aggregates in the sample.

### 2.2. Relationship of Amyloid Fibrils Degradation Induced by Trypsin and Their Polymorphism

**Scheme 1:**
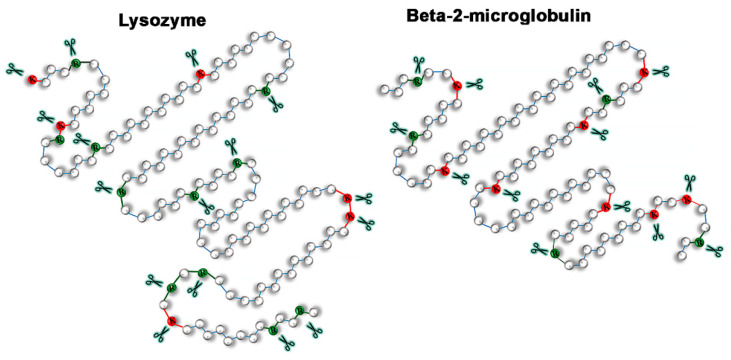
Trypsin cleavage sites in the lysozyme and beta-2-microglobulin amino acid sequences. Lysine (K) and Arginine (R) amino acid residues in the primary sequence of the proteins are highlighted in red and green color, respectively.

Analysis of the lysozyme amino acid sequence indicates that the protein has 17 trypsin cleavage sites located in different parts of the protein (Scheme 1).

Taking into account that the conformation of lysozyme significantly affects its stability, we can suggest that the mechanism of protease action on fibrils may depend on the secondary and tertiary structures of amyloid-forming protein, that is, on different localization of proteolytic cleavage sites. In order to understand this issue, we investigated the effect of protease on lysozyme amyloids formed under alternative conditions (at acidic pH) [30,34].

**Figure 3:**
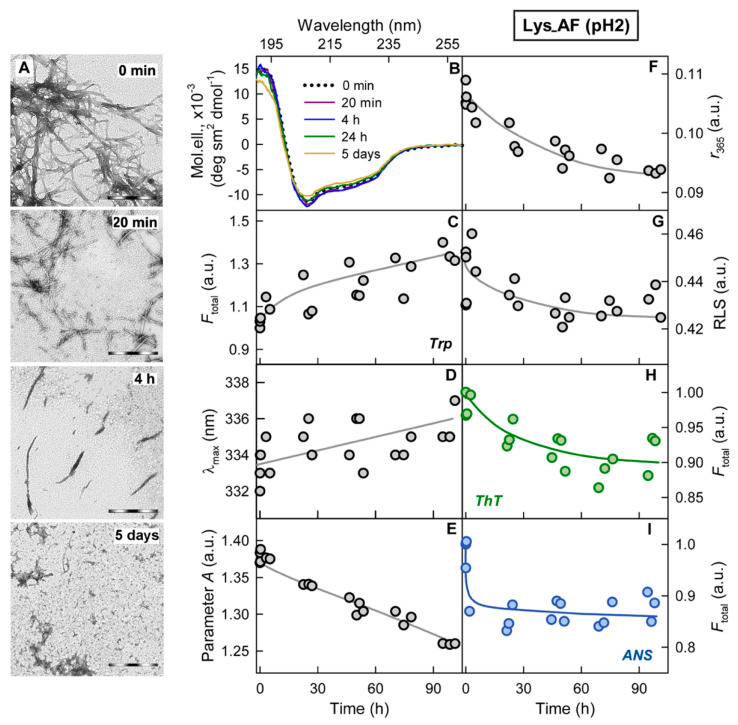
Trypsin-induced degradation of amyloid fibrils formed from lysozyme at pH 2. (**A**) The electron micrographs of amyloid fibrils in the absence and at different time points after the addition of trypsin to the samples. Scale bars are equal to 1 μm. (**B**) Changes in far UV CD spectra of amyloid samples. (**C**) Changes in total fluorescence intensity of amyloid fibrils at the excitation wavelength of 295 nm. (**D**) Changes in wavelength of the fluorescence spectrum maximum of amyloid fibrils (λ_max_). (**E**) Changes in parameter *A* of amyloid fibrils, λ_ex_ = 295 nm. (**F**) Changes in fluorescence anisotropy of amyloid fibrils, λ_ex_ = 295 nm, λ_em_ = 365 nm. (**G**) Changes in Rayleigh Light Scattering of amyloid fibrils. (**H**) Changes in the total fluorescence intensity of ThT and (**I**) ANS in the presence of amyloid fibrils. The values at the panels (**C**,**H**,**I**) are normalized to the values for fibrils in the absence of the protease. The experiments were conducted under physiological conditions (pH 7.4), and weight ratio of fibrils to trypsin was kept at 125:1. The measurements were performed in 20 mM sodium phosphate buffer solution (pH 7.4). The standard error of the mean is determined for a confidence interval of 0.95 and does not exceed 10%.

**Figure 4:**
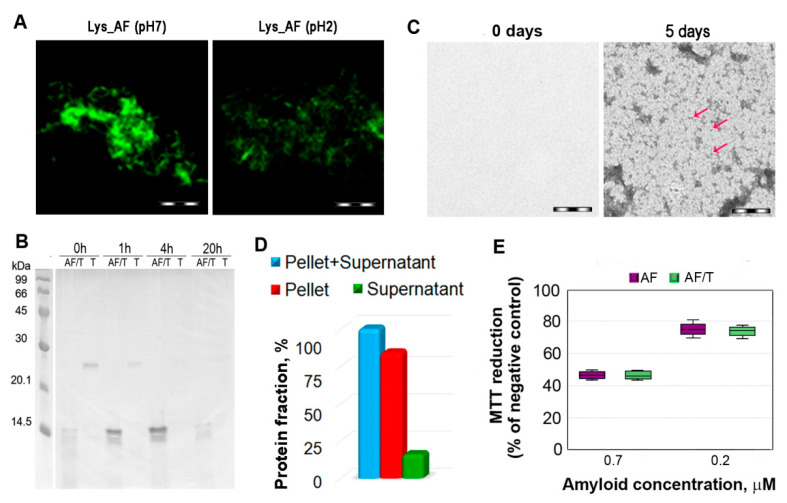
Stability and inhibitory effect on cells of exposed to trypsin amyloid fibrils formed from lysozyme at pH 2. (**A**) The difference in morphology of amyloid fibrils formed from lysozyme at acidic (pH 2) and neutral (pH 7) conditions visualized by confocal microscopy. Scale bars are 3 μm. (**B**) Trypsin-induced degradation of lysozyme amyloid fibrils detected by SDS-PAGE under denaturing conditions. The amyloid fibrils after the addition of trypsin (AF/T) and trypsin (T) were incubated in PBS at 37 °C, and aliquots were taken at different time points. The molecular weight of lysozyme and trypsin is 14.3 and 23.3 kDa, respectively. (**C**) The electron micrographs of supernatant collected by centrifugation of amyloid fibrils before and five days after the addition of trypsin to the sample. Scale bars are equal to 200 nm. The estimated size of aggregates corresponds to 20 (shown by red arrows) or more monomeric protein molecules as evaluated by ImageJ. (**D**) Redistribution of the protein between supernatant and pellet obtained by centrifugation of amyloid fibrils treated with trypsin. (**E**) Dose-dependent inhibition effect of amyloid fibrils on HeLa cells determined by MTT assay. Cells were exposed to amyloid fibrils, intact (AF) or five days after the addition of trypsin (weight ratio of fibrils to trypsin was kept at 7.5/1) (AF/T), at indicated concentrations for 24 h. Data are expressed as medians and interquartile ranges, and whiskers denote 1.5 × IQR. Experiments were repeated at least three times with similar results.

Lysozyme amyloid fibrils prepared under acidic conditions (pH 2.0), as well as fibrils previously prepared under neutral conditions in the presence of GdnHCl, were transferred to a buffer with pH 7.4 and incubated at 37 °C. Previously, it was shown that under these conditions, fibrils retained their structure and stability for a long time [34] that corresponds to our results (Figure S4), which made it possible to observe the change in their characteristics induced by trypsin within a week (Figure 3A–I). The morphology of the prepared amyloid fibrils was analyzed by TEM (Figure 3A). It turned out that lysozyme fibrils, obtained under acidic conditions, were thin long fibers, visually similar to fibrils formed from the same protein at neutral pH of the solution in the presence of denaturant (Figure 1A and Figure 3A). However, TEM data suggested that amyloids prepared under different conditions have different tendencies to clustering. In order to prove this assumption, we analyzed fibril samples using confocal microscopy in the presence of a ThT fluorescent probe. Analysis of confocal microscopy results (Figure 4A), as well as the RLS values of the studied aggregates (Figure 1G and Figure 3G), confirmed our assumption about the smaller size of fibrillar clots in the sample obtained in the acid buffer in comparison to the aggregates formed in the alternative conditions.

It is noteworthy that the values of the parameter *A* and fluorescence anisotropy of lysozyme fibrils prepared at acidic pH (Figure 3E,F) are lower than the values of the corresponding characteristics for lysozyme fibrils obtained at neutral pH (Figure 1E,F). At the same time, the proportion of β-folded structure in lysozyme monomers, which form fibrils prepared at acidic pH, is significantly lower than in fibrils obtained at neutral pH, while the proportion of α-helical elements, on the contrary, is higher (Figure S9A,B). These data indicate that the structure of lysozyme molecules in amyloid fibrils prepared under different conditions is not identical.

The observed polymorphism of lysozyme amyloid fibrils affects their cytotoxicity, which is in good agreement with our earlier data [34,49]. Cell viability in in vitro experiments when cells were treated with lysozyme fibrils prepared at acidic pH turned out to be higher than in the presence of lysozyme fibrils obtained at neutral pH, as evidenced by the difference in the level of reduced MTT in the presence of different types of fibrils by about 20% (Figure 2D and Figure 4E).

The results of our experiments showed that, when trypsin was added to the suspension of fibrils obtained at acidic pH, after 10 min, the enzyme in a soluble form was not detected in the buffer (as in the case of lysozyme fibrils obtained at neutral pH) (Figure S3). This means that all trypsin molecules in the sample are in a fibril-bound state. Thus, the change in the structure of lysozyme fibrils did not break their ability to interact with trypsin.

Next, we examined whether the mechanism of action of trypsin changes with the change of fibrils structure. The experiment was carried out for five days, as in the case of fibrils obtained under alternative conditions. According to TEM data, fragmentation of lysozyme amyloid fibrils formed under acidic conditions begins as early as 20 min after the addition of the protease (that is, earlier than for amyloid fibrils prepared at neutral pH) (Figure 3A). These results are consistent with the data obtained by SDS-PAGE under denaturing conditions (Figure 4B). It turned out that already 20 h after the addition of trypsin to fibrils obtained at acidic pH, a significant decrease in the intensity of the band corresponding to monomeric lysozyme was observed. At the same time, the intensity of this band did not change even after 24 h after adding trypsin to fibrils prepared at neutral pH.

It can be assumed that the lower rate of degradation of lysozyme fibrils obtained at physiological pH is due to a higher degree of clustering, i.e., to a lower availability of proteolytic cleavage sites. Fragmentation of these fibrils with trypsin probably requires preliminary disaggregation of fibrillar clots. However, despite the different rates of degradation, the mechanism of action of trypsin on different types of lysozyme fibrils turned out to be identical: with the time, the fragments of fibrils were “decompacted” and lost their rigid structure. This mechanism of trypsin action was also confirmed by a decrease in the fluorescence intensity of ANS (Figure 3I) and ThT (Figure 3H) in the sample with amyloid fibrils.

It is important to note that the value of the fluorescence intensity of bound dyes (Figure 3H,I), as well as the scale of their RLS (Figure 3G) recorded five days after the addition of trypsin to lysozyme amyloid fibrils prepared under acidic conditions, is comparable in magnitude with the degree of changes in these characteristics obtained for lysozyme amyloid fibrils prepared under neutral conditions. (Figure 1G–I). The proportion of the degraded fibril fraction five days after the addition of trypsin to different types of lysozyme amyloids (Figure 2A and Figure 4C) determined using the samples after centrifugation is also similar (Figure 2B and Figure 4D). It was shown that an increasing of trypsin concentration can lead to differences in proportion of this fraction in the case of different amyloids (Figure S5). At the same time, the analysis of aggregates, formed after the treatment of different types of lysozyme amyloid fibrils with trypsin in different concentrations using pseudo-native SDS-PAGE in 8% polyacrylamide gel [61], showed similar results. Obtained results indicate the absence in the samples of fractions with molecular weights less than 220 kDa (Figure S11).

The results of the assessment of cell viability in the presence of amyloid fibrils indicate that the cytotoxicity of both types of amyloids after their treatment with trypsin (including, in the case of an increase in its concentration by 17 times) practically does not change compared to intact fibrils (Figure 4E), which indicates similar protein species formed as a result of proteolytic cleavage of the studied amyloids, which does not contradict the TEM data (Figure S12).

It is interesting that despite a similar mechanism of lysozyme amyloid fibrils degradation under the action of trypsin, the recorded change in the intrinsic characteristics of tryptophan fluorescence of amyloid fibrils obtained at acidic pH turned out to be less pronounced (Figure 3C–F) than in the case of lysozyme fibrils prepared at neutral pH (Figure 1C–F). In addition, only a slight decrease in the CD signal was observed without a significant change in the shape of the spectrum for amyloid fibrils prepared at acidic pH after the trypsin effect (Figure 3B). However, the assessment of the content of secondary structure elements showed that trypsin’s influence on lysozyme fibrils prepared at acidic pH, as in the case of fibrils obtained at neutral pH, still led to the transformation of the ordered secondary structure of lysozyme into a disordered one (Figure S9B). It can be assumed that the different effect of trypsin on the photophysical properties of lysozyme fibrils obtained under different conditions is due to the different structure of the protein in amyloid fibrils. The absence of a pronounced effect of proteolytic degradation of lysozyme fibrils obtained at acidic pH on the secondary structure of the amyloid-forming protein and the microenvironment of tryptophan amino acid residues in this protein is probably due to the structural features of lysozyme subunits in these fibrils.

As in the case of lysozyme amyloid fibrils prepared at pH 7, for these amyloids, we compared the samples five days after the addition of trypsin at trypsin/protein ratios of 1:125 and 1:1. It turned out that the characteristics of the sample treated with higher concentration of trypsin were significantly closer to the characteristics of the denatured protein. Evaluation of the photophysical characteristics of the protein remained in the supernatant after sedimentation of amyloid fibrils, and large aggregates by centrifugation showed that these characteristics practically coincided with the characteristics of the fully denatured protein (Figure S8).

Based on the obtained results, it was concluded that polymorphism of amyloid fibrils originating from the same protein (differing in the degree of clustering, secondary and tertiary structures of the protein in amyloid fibrils) could affect the rate of trypsin-induced degradation of amyloid fibrils but had no impact on the mechanism of this process and the cytotoxicity of degraded amyloid aggregates. The discovered relationship between the structure of fibrils and the rate of their degradation by trypsin can become the basis for the development of a new express method for the analysis of amyloid polymorphism.

### 2.3. Relationship of Amyloid Fibrils Degradation Induced by Trypsin and Primary Structure of Amyloid-Forming Protein

In order to determine whether the amino acid sequence of the amyloidogenic protein affects the mechanism of trypsin action, we prepared amyloid fibrils on the basis of another amyloidogenic protein, beta-2-microglobulin, the accumulation of which leads to the development of dialysis-related amyloidosis (DRA, [29]). Analysis of the primary structure of beta-2-microglobulin indicates that the protein has 13 trypsin cleavage sites located in different parts of the protein (Scheme 1). SDS-PAGE results of beta-2-microglobulin sample after adding trypsin indicate that the band corresponding to the monomeric protein is noticeably weakened within 30 min after the addition of protease to the sample, and after 24 h it is no longer detected (Figure S7). Thus, it was shown that beta-2-microglobulin in native state, in contrast to lysozyme, was not resistant to the action of trypsin.

**Figure 5:**
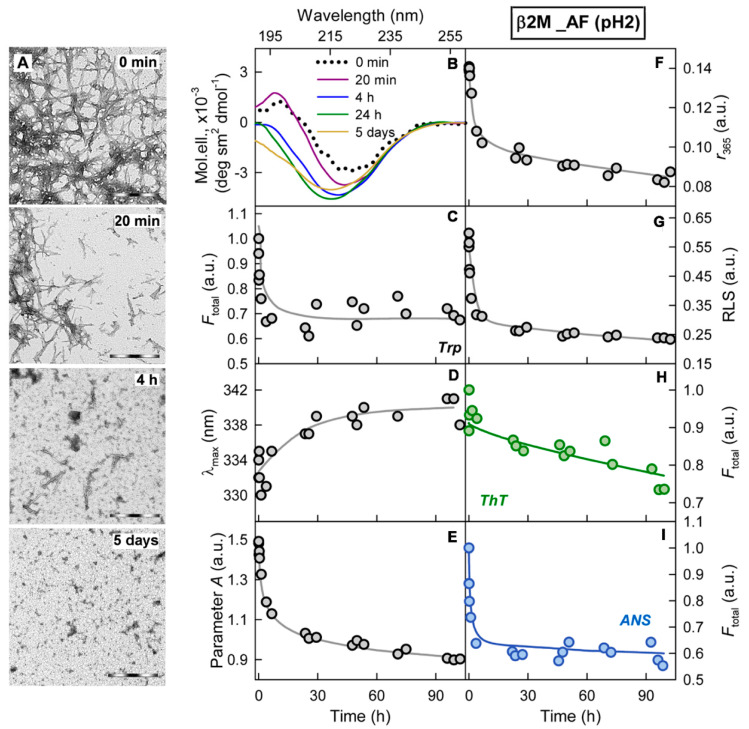
Trypsin-induced degradation of amyloid fibrils formed from beta-2-microglobulin (β2M) at pH 2. (**A**) The electron micrographs of amyloid fibrils in the absence (Partly Reprinted from ref. [34]) and at different time points after the addition of trypsin to the samples. Scale bars are equal to 1 μm. (**B**) Changes in far UV CD spectra of amyloid samples. (**C**) Changes in total fluorescence intensity of amyloid fibrils at the excitation wavelength of 295 nm. (**D**) Changes in wavelength of the fluorescence spectrum maximum of amyloid fibrils (λ_max_). (**E**) Changes in parameter A of amyloid fibrils, λ_ex_ = 295 nm. (**F**) Changes in fluorescence anisotropy of amyloid fibrils, λ_ex_ = 295 nm, λ_em_ = 365 nm. (**G**) Changes in Rayleigh Light Scattering of amyloid fibrils. (**H**) Changes in the total fluorescence intensity of ThT and (**I**) ANS in the presence of amyloid fibrils. The values at the panels (**C**,**H**,**I**) are normalized to the values for fibrils in the absence of the protease. The experiments were conducted under physiological conditions (pH 7.4) and weight ratio of fibrils to trypsin was kept at 125:1. The measurements were performed in 20 mM sodium phosphate buffer solution (pH 7.4). The standard error of the mean is determined for a confidence interval of 0.95 and does not exceed 10%.

Beta-2-microglobulin amyloid fibrils were prepared under acidic conditions (pH 2.0), like lysozyme fibrils, after which they were transferred to neutral conditions and incubated at 37 °C as a control (Figure S4). As in the case of previously studied amyloid fibrils, the structure and stability of the prepared protein aggregates after the adding of trypsin were analyzed using a wide range of physicochemical approaches (Figure 5A–I). The morphology of the prepared amyloid fibrils was analyzed by TEM (Figure 5A). It turned out that beta-2-microglobulin fibrils in their morphology and degree of clustering were more similar to lysozyme amyloid fibrils obtained under acidic conditions (Figure 3A and Figure 5A) than to lysozyme fibrils obtained under neutral conditions (Figure 1A). This was also confirmed by the RLS value of beta-2-microglobulin amyloids (Figure 5G). At the same time, the content of β-strands that form the fibril core in the sample with beta-2-microglobulin fibrils turned out to be rather high (closer to the values observed for lysozyme amyloid fibrils obtained at neutral pH in the presence of GdnHCl) (Figure S9C). Thus, the morphology of beta-2-microglobulin fibrils is more similar to lysozyme fibrils obtained at acidic pH, and their secondary structure is more similar to lysozyme fibrils prepared under neutral conditions.

**Figure 6:**
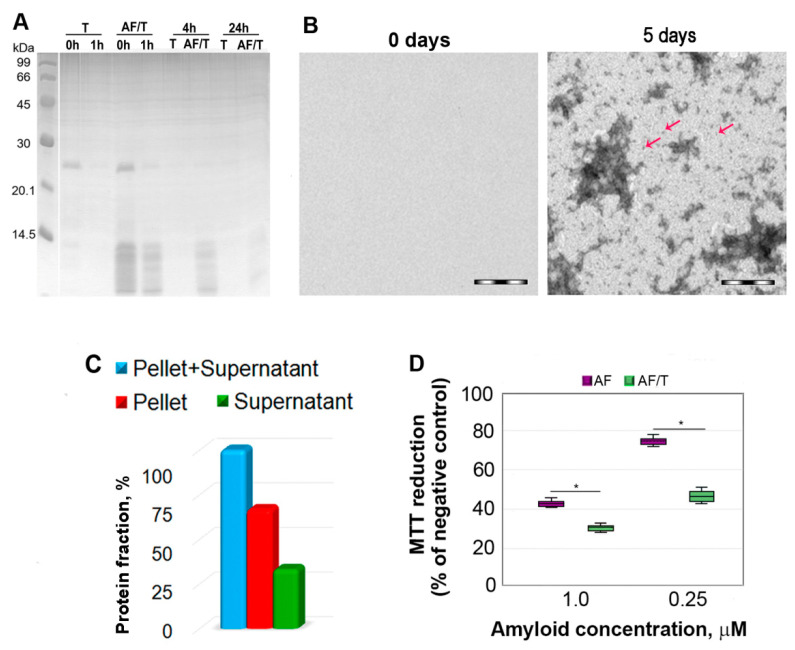
Stability and inhibitory effect on cells of exposed to trypsin amyloid fibrils formed from beta-2-microglobulin (β2M) at pH 2. (**A**) Trypsin-induced degradation of beta-2-microglobulin amyloid fibrils detected by SDS-PAGE under denaturing conditions. The amyloid fibrils after adding trypsin (AF/T) and trypsin (T) solutions were incubated in PBS at 37 °C, and aliquots were taken at different time points. Molecular weight of beta-2-microglobulin and trypsin is 11.8 and 23.3 kDa, respectively. (**B**) The electron micrographs of supernatant collected by centrifugation of amyloid fibrils before and five days after adding trypsin. Scale bars are equal to 200 nm. The estimated size of aggregates corresponds to 20 (shown by red arrows) or more monomeric protein molecules as evaluated by ImageJ. (**C**) Redistribution of the protein between supernatant and pellet obtained by centrifugation of amyloid fibrils treated with trypsin. (**D**) Dose-dependent inhibition effect of amyloid fibrils on HeLa cells determined by MTT assay. Cells were exposed to amyloid fibrils, intact or treated with trypsin (weight ratio of fibrils to trypsin was kept at 7.5/1), at indicated concentrations for 24 h. Data are expressed as medians and interquartile ranges, whiskers denote 1.5 × IQR. Experiments were repeated at least three times with similar results. ANOVA, Tukey post hoc test, * *p* < 0.01.

According to TEM data, fragmentation of beta-2-microglobulin amyloid fibrils begins as early as 20 min after adding trypsin (as in the case of lysozyme fibrils formed at acidic pH) (Figure 5A). These results are in good agreement with the data obtained by SDS-PAGE under denaturing conditions (Figure 6A). A significant decrease in the intensity of the band of monomeric beta-2-microglobulin was observed already 24 h after the addition of trypsin, as in the case of lysozyme fibrils obtained under the same conditions. In contrast, in the case of lysozyme fibrils prepared at neutral pH, the intensity of the band of monomeric lysozyme did not change significantly 24 h after the addition of trypsin. The obtained results confirm the previously made the assumption that the rate of amyloid fibrils degradation is influenced by their morphology and the degree of clustering.

SDS-PAGE of amyloid fibrils formed from beta-2-microglobulin untreated with trypsin showed a band with a molecular weight of 23–24 kDa, corresponding to the molecular weight of the dimeric form of the protein (Figure S13). SDS-PAGE of the sample of beta-2-microglobulin fibrils mixed with trypsin, the proteolytic activity of which was quenched by PMSF, revealed two separate bands with close molecular weights (about 23–24 kDa), corresponding to the molecular weight of beta-2-microglobulin dimer found in amyloid fibrils untreated with trypsin, and to the molecular weight of trypsin (Figure S13). Beta-2-microglobulin dimer exhibited a lower electrophoretic mobility compared to trypsin. SDS-PAGE of amyloid beta-2-microglobulin fibrils treated with trypsin showed a single band with a molecular weight of 23–24 kDa (Figure 6A), corresponding to the molecular weight of trypsin, and not to the dimeric form beta-2-microglobulin (which appears to have been degraded immediately after the addition of trypsin). Thus, in the case of beta-2-microglobulin fibrils in contrast to lysozyme fibrils, trypsin can be detected in the sample using SDS-PAGE.

TEM data indicate that, after adding trypsin to the sample, the fibril fragments lose their rigid fiber structure, as in the case of various types of lysozyme amyloid fibrils (Figure 5A and Figure S12). This mechanism of trypsin action is also confirmed by a decrease in the fluorescence intensity of ANS (Figure 5I) and ThT (Figure 5H) in the sample. Thus, the mechanism of action of trypsin is not affected by either the morphology or the structure (primary and secondary) of the amyloidogenic protein.

The trypsin-induced change in the fluorescence intensity of the dyes bound to beta-2-microglobulin amyloid fibrils (Figure 5H,I), and the decrease in the RLS value measured for them five days after the start of the experiment (Figure 5G) were more pronounced than the change in these characteristics for both types of lysozyme amyloid fibrils (Figure 1G–I and Figure 5G–I). In addition, the proportion of the degraded fraction of fibrils five days after the addition of trypsin in the case of beta-2-microglobulin amyloids (Figure 2A, Figure 4C and Figure 6B) was more than 30% and was noticeably higher than in the case of both types of lysozyme amyloid fibrils (Figure 2B, Figure 4D and Figure 6C). This indicates that the efficiency of proteolytic degradation of amyloid fibrils depends on the primary structure of the protein.

After treatment of beta-2-microglobulin amyloid fibrils with trypsin, a significant decrease in the values of parameter *A* (Figure 5E) and fluorescence anisotropy (Figure 5F) was observed, as well as a red shift in the maximum of the fluorescence spectrum of the sample (Figure 5D). In addition, a significant decrease in the integral fluorescence intensity of tryptophan residues of amyloid fibrils (Figure 5C) after adding trypsin was found. The shape of the CD spectrum of beta-2-microglobulin amyloid fibrils differed from the shape of the CD spectrum of the sample measured 20 min after adding trypsin (Figure 5B). The change in the shape of the CD spectrum of the sample recorded 24 h after adding trypsin was even more pronounced. According to the assessment of the content of various types of secondary structure in the samples, the trypsin addition to the studied amyloid fibrils leads to an increase in the proportion of disordered structure due to a decrease in the proportion of β-strands in the amyloid-forming protein (Figure S9C). The observed changes confirm the assumption about the degradation of amyloid fibrils.

Trypsin can be detected in a sample with beta-2-microglobulin amyloid fibrils no more than a day after its addition by SDS-PAGE (Figure 6A). The most pronounced changes in the photophysical characteristics and morphology of these amyloid fibrils after trypsin adding were observed in the same period (Figure 5). Further moderate changes either may be associated with the immediate action of a small amount of trypsin molecules remaining in the sample, which avoid detection by SDS-PAGE, or may be the result of processes triggered by trypsin on the first day after its addition (leading to the decreased stability of fibrils).

The results obtained in the study of the cytotoxicity of beta-2-microglobulin amyloid fibrils after trypsin addition were quite unexpected. We not only did not observe a decrease but observed an increase in the cytotoxicity of amyloids of this protein after their treatment with trypsin (Figure 6D). It can be assumed that, in the case of beta-2-microglobulin fibrils, the cytotoxicity of the forming amyloid aggregates (fragments of amyloid fibrils or non-fibrillar amyloid aggregates) is higher than in the case of amyloid fibrils of lysozyme. Another reason for the observed effect may be the greater number of formed toxic non-fibrillar aggregates (Figure S12) in relation to non-toxic protein monomers and their fragments (Figure S10) than in the case of lysozyme fibrils.

Thus, the results obtained on the example of two amyloidogenic proteins (and three polymorphic structures) made it possible to assume that the mechanism of fibril degradation under the action of trypsin did not depend on the morphology or primary and secondary structure of the amyloidogenic protein. At the same time, the rate of proteolytic degradation of amyloids depends on the morphology and degree of clustering of fibrils. In addition, it was suggested that the cytotoxicity of trypsin-degraded amyloid fibrils might depend on the ratio of highly toxic and low toxic/non-toxic protein species in the sample after such exposure.

## 3. Discussion

In this study, we demonstrated that the proteolytic enzyme of the pancreas, trypsin, can lead to degradation of amyloid fibrils, which results in fragmentation of their fiber and a decrease in the ordering of amyloid-forming proteins with the formation of non-fibrillar aggregates of various sizes. In this case, trypsin facilitates the degradation of fibrils across the entire length of their fiber rather than starting from the fibril ends, suggesting that proteolysis is not directional. These ideas are in good agreement with the results of the work Chander et al. [19], where through the electron microscopic analysis of immunogold labeling of trypsin binding to Aβ40 and Aβ42, the protease binding to fibrils along the entire length of their fiber and not only to the ends of the fibrils was shown. It should be noted that the mechanism of proteolytic degradation of amyloid fibrils observed by us is similar to that previously proposed by Poepsel et al. [17] in the study of fragmentation of the tau protein fibrils under the action of serine protease HTRA1 (belongs to the high temperature requirement A (HtrA) family of serine proteases), implying the universality of protease action. According to the observed degradation mechanism, trypsin can affect both the structure of protein molecules forming β-sheets and intermolecular hydrogen bonds involved in the formation of amyloid fibers.

Our data for two amyloidogenic proteins (and three polymorphic structures) show the reliance of the effectiveness and rate of trypsin action on amyloid fibrils on the structure of the amyloid-forming protein (primary, secondary, and tertiary), which determines the number and localization of proteolytic cleavage sites, as well as on the morphology and degree of clustering of amyloid fibrils, which determine the availability of trypsin binding sites. This is in line with the literature data [17], indicating that the disintegration activity of HTRA1 significantly increases the rate of proteolytic degradation of densely packed areas of fibrils, which leads to more efficient fragmentation of amyloid fibers.

Binding of proteases to amyloids and proteolytic degradation of fibrils, at first glance, seems to be a positive effect in terms of amyloidosis treatment; however, this is actually not entirely true. The interaction of trypsin with abnormal aggregates amyloid fibrils, which was shown in our work, as well as in the work [19], can lead to the inability of the enzyme interaction with other substrates. For example, trypsin is required for Aβ-peptide catabolism, and its interaction with Aβ-peptide amyloids can reduce clearance of soluble Aβ-peptide, and an increased concentration of Aβ-peptide enhances its aggregation. A similar conclusion was reached in the study of the trypsin binding to fibrillar Aβ-peptide [19]. This assumption is also in a good agreement with the results according to which mice deficient of proteolytic enzymes exhibited elevated levels of Aβ while overexpression of neprilysin in APP-transgenic mice could reduce the deposition of fibrillar Aβ [62,63].

In addition, we showed that proteolytic degradation of amyloids did not reduce cytotoxicity of the protein aggregates for Hela cells, as might be expected. We attribute this to the formation of highly toxic non-fibrillar aggregates along with the formation of non-toxic protein monomers and their fragments induced by trypsin. A high cytotoxicity of large disordered aggregates forming after exposure of the lysozyme and beta-2-microglobulin fibrils to the protein with chaperone activity alpha-B-crystallin was already demonstrated by us earlier [34].

In conclusion, here, we show that the proteolytic enzyme of the pancreas, trypsin, can induce degradation of amyloid fibrils, and the mechanism of this process is qualitatively the same for amyloids with different structure and morphology. At the same time, the efficiency and rate of fibril degradation are dependent on the structure of the amyloid-forming protein as well as on the morphology and clustering of amyloid fibrils, which determines the number, localization, and availability of protease cleavage sites. Proteolytic degradation of amyloids not only does not reduce, as might be expected, but, in some cases, even increases cytotoxicity of the amyloid fibrils, presumably due to the formation of more toxic non-fibrillar aggregates.

## 4. Materials and Methods

### 4.1. Materials

Guanidine hydrochloride (GdnHCl), lysozyme, buffer components, trypsin from bovine pancreas, and 3-(4,5-dimethylthiazol-2-yl)-2,5-diphenyltetrazolium bromide (MTT) were purchased from Sigma (St. Louis, MO, USA). Thioflavin T (ThT) “UltraPure Grade” was supplied by AnaSpec (Fremont, CA, USA), and 8-anilino-1-naphthalene sulfonate (ANS) was from Serva (Heidelberg, Germany). Further purification of chemicals has not been performed. Reagents for cell cultivation, including DMEM medium (glucose 4.5 g/L), fetal bovine serum (FBS), and 0.25% Trypsin-EDTA were acquired from Gibco (Thermo Fisher Scientific, Waltham, MA, USA). Culture flasks and 96-well plates (flat bottom) were from Corning (New York, NY, USA).

### 4.2. Amyloid Fibrils Preparation

For the preparation of amyloid fibrils from beta-2-microglobulin, the protocol described in the work [64] was used. The samples of recombinant beta-2-microglobulin were received from Mikhail M. Shavlovsky, Dmitry S. Polyakov, and Rodion G. Sakhabeev (Department of Molecular Genetics, Institute of Experimental Medicine). Lysozyme amyloid fibrils were grown under the following conditions: (1) 100 mM KH_2_PO_4_/NaOH (pH 7) supplemented with 3 M GdnHCl and incubation at 57 °C, and (2) 20% acetic acid/100 mM NaCl (pH 2) and incubation at 37 °C [30]. The concentration of a protein was 2 mg/mL. Fibrilogenesis was performed with constant agitation for one day (500 rpm). The constant temperature during fibril grows, maintained by the TS-100 Thermo-Shaker (Biosan, Warren, MI, USA). A two-step buffer exchange in mature amyloid fibrils (with deionized water and then with 20 mM sodium phosphate buffer (pH 7.4)) was made by dialysis.

### 4.3. Amyloid Fibrils Degradation by Trypsin

The final concentration of fibril used for spectroscopic measurement was 0.15 mg/mL. To avoid autolysis [31,65], trypsin stock was stored frozen in acidic conditions (1 mM HCl); it was thawed immediately before adding to the fibril solutions (the weight ratio of enzyme to fibrils was 1 to 125 unless otherwise specified). In some cases, the concentration of trypsin was increased so that the ratio of protease to fibril was 1:7.5 and 1:1.

The experiment was initiated by manual mixing of the fibril solutions with protease, and the measurements were carried out within five days. The dead-time of measurements with manual mixing was estimated as 4 s. [38,39] TEM images were acquired at different time points sampling 10 μL of fibril/trypsin mixture.

To prepare samples for SDS-PAGE under denaturing conditions, the concentration of amyloid fibrils increased up to 24 mg/mL, and trypsin concentration was 0.4 mg/mL. Thus, the weight ratio of the protease to amyloid fibrils was 1:60. The protease in this concentration can be detectable by 17% SDS-PAGE. For identification of aggregates formed after treatment of amyloid fibrils with trypsin, we modified the experiment as follows: samples of amyloid fibrils five days after the addition of trypsin in a ratio of 7.5:1 and 1:1 were analyzed using an 8% polyacrylamide gel.

In the experiments on resistance of the native proteins to proteolytic cleavage by trypsin, the following conditions were used: the concentration of native lysozyme and beta-2-microglobulin was kept at 0.4 mg/mL, and the weight ratio of both proteins to protease was 10:1.

Proteolytic digestion of native bovine serum albumin (BSA) by trypsin was performed at 37 °C. Aliquots of reaction mixture were collected at different times with the addition of phenylmethylsulfonyl fluoride (PMSF) to stop the proteolysis. The concentration BSA was kept at 50 mg/mL, and weight ratio of the protein to protease was 125:1.

### 4.4. Transmission Electron Microscopy

A transmission electron microscope Libra 120 (Carl Zeiss, Oberkochen, Germany) was applied to produce micrograph amyloid fibrils and degraded amyloid aggregates after proteases exposure. Samples were put on the cooper grids coated with formvar/carbon films (Electron Microscopy Sciences, Hatfield, PA, USA) and stained by a 1% aqueous solution of uranyl acetate. TEM images were analyzed using ImageJ software. The number of monomers comprising the aggregates was estimated by their area.

### 4.5. Confocal Microscopy

Confocal laser scanning microscope Olympus FV 3000 (Olympus, Tokyo, Japan) and oil immersion objective with a 60× magnification, numerical aperture NA 1.42, and laser with excitation line 405 nm was applied to reveal the difference in morphology of ThT-stained amyloids formed from lysozyme in the different conditions.

### 4.6. Spectral Measurements

The samples prepared according to the Section 4.3 were used for fluorescence studies. A U-3900H spectrophotometer (Hitachi, Tokyo, Japan) was applied to collect the absorption spectra of the samples. The absorption spectra of amyloid fibrils and mixtures of fibrils with ThT or ANS were corrected by the light scattering according to standard procedure [66]. The concentration of ThT and ANS and fibrils from lysozyme and beta-2-microglobulin was quantified using molar extinction coefficients of *ε*_412_ = 31,600 M^−1^ cm^−1^, *ε*_350_ = 5000 M^−1^ cm^−1^, *ε*_280_ = 36,000 M^−1^ cm^−1^, and *ε*_276_ = 20,065 M^−1^ cm^−1^, respectively. The absorbance of a dye did not exceed 0.5 in experiments with ThT and ANS.

Fluorescence spectra of samples was measured using the Cary Eclipse spectrofluorimeter (Varian, Melbourne, Australia). The parameters of tryptophan fluorescence of the fibrils were recorded using excitation wavelength of 295 nm and corrected for instrument sensitivity. The change of the position and shape of the fluorescence spectra of fibrils was tested by parameter *A* = *I*_320_*/I*_365_, with *I*_320_ and *I*_365_ being the fluorescence intensities at the emission wavelengths of 320 and 365 nm, respectively [38,39]. The anisotropy of tryptophan fluorescence was calculated by the equation:

(1) $$r=\frac{\left(I_{V}^{V}-GI_{H}^{V}\right)}{\left(I_{V}^{V}+2GI_{H}^{V}\right)}$$

where $I_{V}^{V}$ and $I_{H}^{V}$ are vertical and horizontal components of the fluorescence intensity excited by vertically polarized light and recorded at the wavelength of 365 nm, and $G=I_{V}^{H}/I_{H}^{H}$ is the coefficient that takes into account that instrument sensitivity for the vertical and horizontal components of the fluorescence is different [67]. The size of aggregates in the fibril samples was characterized by Rayleigh light scattering (RLS) recorded using the same wavelength of excitation and registration (295 nm). Trypsin molecules themselves had a low RLS (close to RLS of buffer solution alone) due to the low concentration of proteases used.

Fluorescence of ThT and ANS was excited at a wavelength of 440 and 350 nm, respectively. The spectral slits width did not exceed 5 nm in most experiments. Increasing the slit widths did not influence the experimental results. The fluorescence intensity was corrected on the primary inner filter effect [68]. The fluorescence intensity of ThT and ANS in the presence of trypsin without amyloids was equal to that of the dyes buffered solutions and did not contribute to the ThT and ANS intensities during the degradation of amyloid fibrils.

Far-UV CD spectra (190–260 nm) of fibrils were measured using a J-810 spectropolarimeter (Jasco, Tokyo, Japan) equipped with a 1 mm path length cell. Three scans of CD spectra were averaged and base line corrected, which was achieved using the appropriate buffer. The CD spectrum of the trypsin in the concentration used matches the signal of the buffer solution alone. CD spectra of fibrils were corrected for CD spectrum of the trypsin. The secondary structure content of fibrils was estimated from their corrected CD spectra, which were used to determine in the CDPro package [58] that incorporates three regression methods (Selcon, Contin, and CDSSTR) and several basic sets of proteins (including soluble, membrane, and denatured proteins with a known secondary structure).

### 4.7. MTT Assay

HeLa cells were propagated in DMEM medium supplemented with 10% FBS, 50 μg/mL penicillin-streptomycin, and 2 mM l-glutamine and were grown in a humidified incubator with 5% CO_2_ at 37 °C. A day before the MTT assay, confluent HeLa cells were seeded in 96-well coated culture plates at density of 3000 viable cells/well in 120 µL of culture medium and were kept at at 37 °C and 5% CO_2_. Amyloid aggregates were added to cells at a final concentration of 0.7 μM (lysozyme fibrils) or 1 μM (beta-2-microglobulin fibrils). The weight ratio of protein to trypsin was 125:1. Additionally, to enrich the samples of amyloid fibrils with the degraded fraction, for this experiment, we increased the concentration of trypsin 17-fold, so the weight ratio of protein to trypsin was 7.5:1. The same mass ratio of protein and trypsin (7.5:1) was used to obtain fragments of monomeric lysozyme, which was preliminarily boiled for 30 min to transform the protein (that is highly protease-resistant in native state) into a denatured state. The toxicity of aggregates to cell was evaluated 24 h after their addition to cells by the MTT (Sigma-Aldrich, St. Louis, MO, USA) reduction inhibition assay according to standard protocol [69,70] using an automatic plate reader (Bio-Rad, Milan, Italy). Cell viability was assessed based on the value of the absorption of MMT dye. The absorption of MMT dye was the average of five independent measurements as corrected for the blank. Recorded data were normalized to the value for controls containing equal amounts of trypsin in amyloid incubation buffer.

### 4.8. Statistical Analysis

The spectral data of fibrils are shown as the mean of at least three independent measurements. The data of MMT assay are demonstrated as the median of three experiments.

Statistical analysis performed using GraphPad Prism (Version 9.1.0) software (GraphPad Software, San Diego, California, USA). The reliability of the results was verified using Student *t*-test for paired measurement and one-way analysis of variance (ANOVA) for unpaired measurement.

## Supplementary Materials

The following are available online at https://www.mdpi.com/1422-0067/22/9/4828/s1: Figure S1: Autolysis of trypsin in PBS at 37 °C, Figure S2: Trypsin digestion of native bovine serum albumin (BSA), Figure S3: The binding of trypsin to lysozyme amyloid fibrils prepared at pH2 and pH7, Figure S4: The stability of amyloid fibrils, Figure S5: Redistribution of the protein between supernatant and pellet obtained by centrifugation of amyloid fibrils formed from lysozyme at pH 7 (Lys_AF (pH 7)) and at pH 2 (Lys_AF (pH 2)) treated with trypsin, Figure S6: Trypsin induced degradation of lysozyme amyloid fibrils prepared at pH7, Figure S7: Trypsin digestion of lysozyme and beta-2-microglobulin (β2M) in their native state, Figure S8: Spectral characteristics of amyloid fibrils formed from lysozyme at pH7 and at pH2 treated with trypsin, Figure S9: Trypsin induced changes of the secondary structure of amyloid fibrils formed from lysozyme at pH7, lysozyme at pH2 and beta-2-microglobulin at pH2, Figure S10: The effect of amyloidogenic proteins and their fragments on the cells determined by MTT assay, Figure S11: Identification of aggregates formed after treatment of amyloid fibrils with trypsin, Figure S12: Characteristics of aggregates formed after treatment of amyloid fibrils with trypsin, Figure S13: SDS-PAGE of beta-2-microglobulin amyloid fibrils mixed with trypsin.
