# Correction: Stepanenko et al. Trypsin Induced Degradation of Amyloid Fibrils. *Int. J. Mol. Sci.* **2021**, *22*, 4828

**DOI:** 10.3390/ijms25126293

**Published:** 2024-06-07

**Authors:** Olga V. Stepanenko, Maksim I. Sulatsky, Ekaterina V. Mikhailova, Olesya V. Stepanenko, Irina M. Kuznetsova, Konstantin K. Turoverov, Anna I. Sulatskaya

**Affiliations:** 1 Laboratory of Structural Dynamics, Stability and Folding of Proteins, Institute of Cytology, Russian Academy of Sciences, 4 Tikhoretsky Avenue, 194064 St. Petersburg, Russia; sov@incras.ru (O.V.S.); 4evamkh@gmail.com (E.V.M.); lvs@incras.ru (O.V.S.); imk@incras.ru (I.M.K.); ansul@mail.ru (A.I.S.); 2 Laboratory of Cell Morphology, Institute of Cytology, Russian Academy of Sciences, 4 Tikhoretsky Avenue, 194064 St. Petersburg, Russia; m_sulatsky@mail.ru; 3 Institute of Physics, Nanotechnology and Telecommunications, Peter the Great St. Petersburg Polytechnic University, Polytechnicheskaya 29, 195251 St. Petersburg, Russia

In the original [1] and in our earlier publication [2], the control sample of beta-2-microglobulin amyloids before their treatment with active substances (trypsin and alpha-B-crystalline) was the same. Therefore, the micrographs at time zero (Figure 5A in [1] and Figure 1 in [2]) partially overlap. The reference [2] is the reference [34] of the publication [1]. And the new reference citation to the caption of Figure 5A of the publication [1] and information about this overlapping has now been inserted.

The citation has now been inserted in Figure 5 caption and should read:

“(**A**) The electron micrographs of amyloid fibrils in the absence (Partly Reprinted from ref. [34]) and at different time points after the addition of trypsin to the samples.”

The authors state that the scientific conclusions are unaffected. This correction was approved by the Academic Editor. The original publication has also been updated.
