# Supplementary material for: Trypsin Induced Degradation of Amyloid Fibrils
Source: Int J Mol Sci. 2021 May 2;22(9):4828. doi: 10.3390/ijms22094828 (PMC8124345; doi:10.3390/ijms22094828)
Supplement: Supplementary file 1 [file ijms-22-04828-s001.zip › ijms-1188959-supplementary.pdf]

SUPPLEMENTARY INFORMATION

Trypsin induced degradation of amyloid fibrils

Olga V. Stepanenko[1], M.I. Sulatsky[2], E.V. Mikhailova[1], Olesya V. Stepanenko[1], I. M. Kuznetsova[1], K. K. Turoverov[1, 3, *] and A.I. Sulatskaya[1]

[1] Laboratory of structural dynamics, stability and folding of proteins, Institute of Cytology, Russian Academy of Sciences, 4 Tikhoretsky ave., St. Petersburg 194064, Russian Federation;
[2] Laboratory of cell morphology, Institute of Cytology, Russian Academy of Sciences, 4 Tikhoretsky ave., St. Petersburg 194064, Russian Federation;
[3] Peter the Great St.-Petersburg Polytechnic University, St. Petersburg, Polytechnicheskaya 29, 195251, Russia

sov@incras.ru (O.V.S.); m_sulatsky@mail.ru (M.I.S.); 4evamkh@gmail.com (E.V.M.); lvs@incras.ru (O.V.S.); imk@incras.ru (I.M.K.); ansul@mail.ru (A.I.S.)

[*]Corresponding author: K.K. Turoverov, e-mail: kkt@incras.ru, Tel.: +7 812 297 19 57, Fax: +7 812 297 35 41

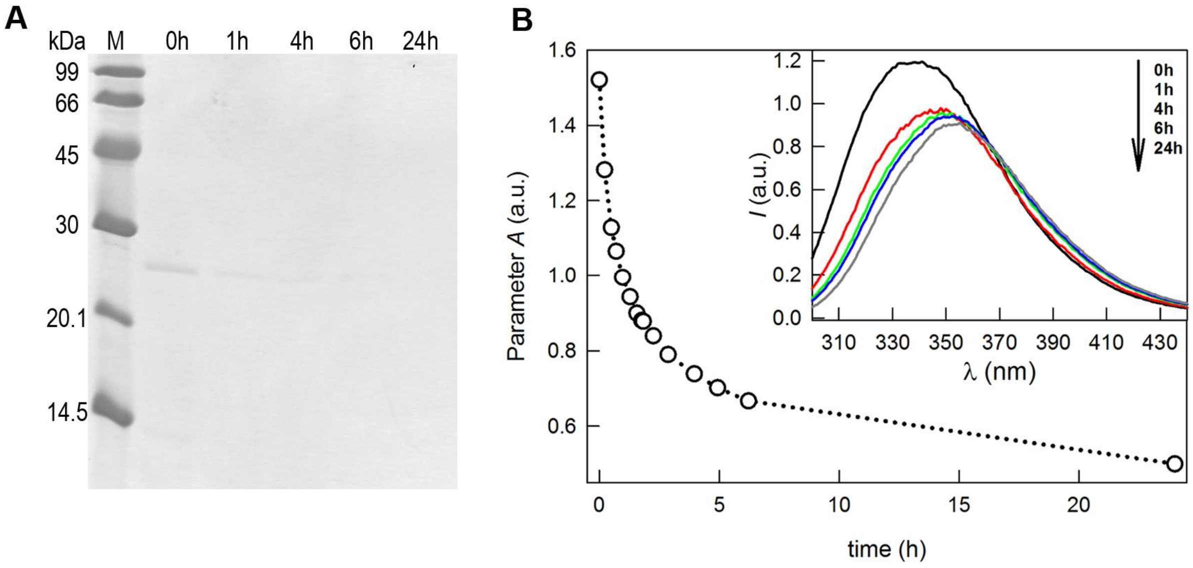

Figure S1. Autolysis of trypsin in PBS at 37 °C. (A) SDS-PAGE under denaturing conditions of trypsin samples collected at different time points and loaded into the wells as follows: freshly thawed (0h), and after 1, 4, 6 and 24h incubation at 37 °C. (B) Temporal changes in parameter *A* of trypsin incubated at 37 °C, λ_{ex} = 295 nm. The inset demonstrates the changes in tryptophan fluorescence spectrum of trypsin at the starting point (0h, black curve) and 1 (red curve), 4 (green curve), 6 (blue curve) and 24 (gray curve) hours after the start of the experiment. Protease concentration was 0.3 mg/ml.

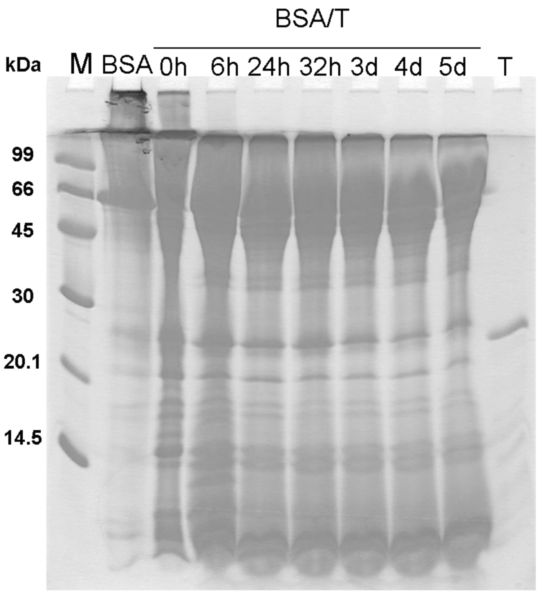

Figure S2. Trypsin digestion of native bovine serum albumin (BSA). Protein at high concentration (50 mg/ml) untreated with trypsin and at different time points after the addition of trypsin (trypsin/protein ratios of 1:125) incubated in PBS at 37 °C were loaded on SDS-PAGE under denaturing conditions. Freshly prepared trypsin (T) at concentration 0.4 mg/ml was loaded as well. Molecular weight of BSA is 66.5 kDa.

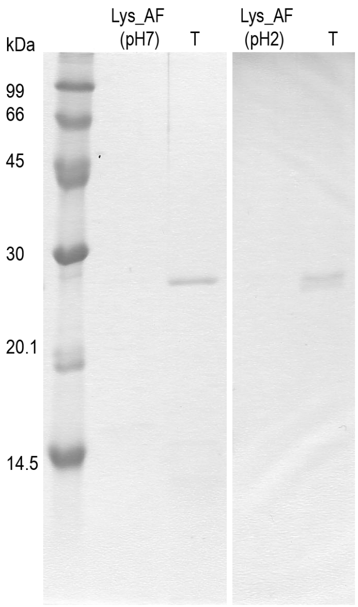

Figure S3. The binding of trypsin to lysozyme amyloid fibrils prepared at pH2 and pH7. The samples were prepared as follows: amyloid fibrils in the presence of trypsin and trypsin alone were incubated in PBS for 10 min at 37 °C. The trypsin was than inactivated with PMSF and samples were centrifuged at high speed for 1 hour. Supernatants of lysozyme amyloid fibrils prepared at pH7 (Lys_AF (pH7)) and pH2 (Lys_AF (pH2)) followed by the addition of trypsin, and trypsin alone (T) were collected and loaded into the wells. Molecular weight of lysozyme and trypsin is 14.3 and 23.3 kDa, respectively.

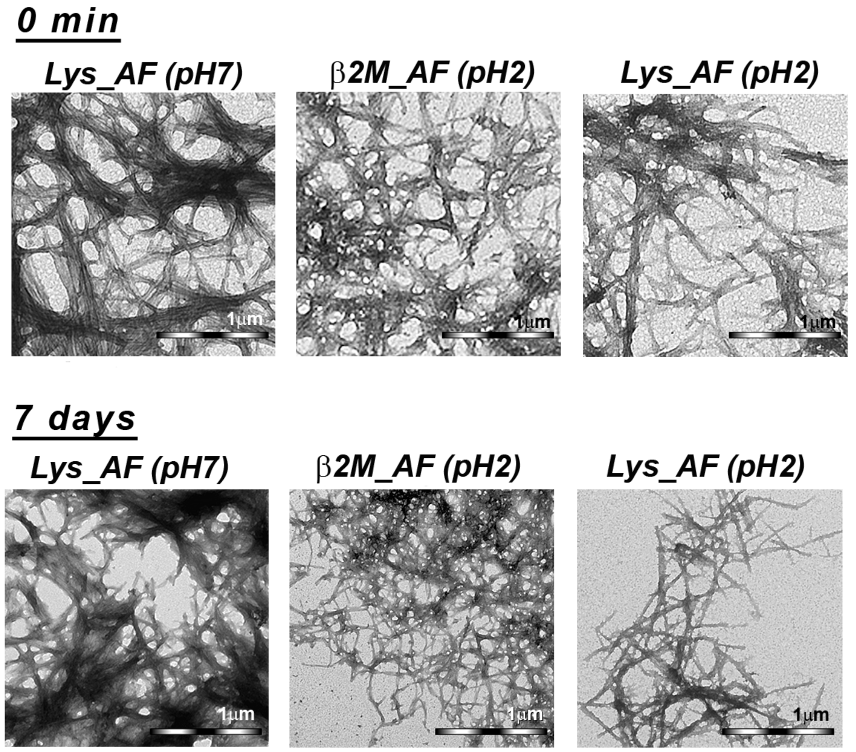

Figure S4. The stability of amyloid fibrils. The electron micrographs of the intact amyloids formed from lysozyme at pH7 and at pH2 and from beta-2-microglobulin at pH2 were collected at the start (0 min) and after 7 days incubation at 37 °C (7 days). Scale bars are equal to 1 μm.

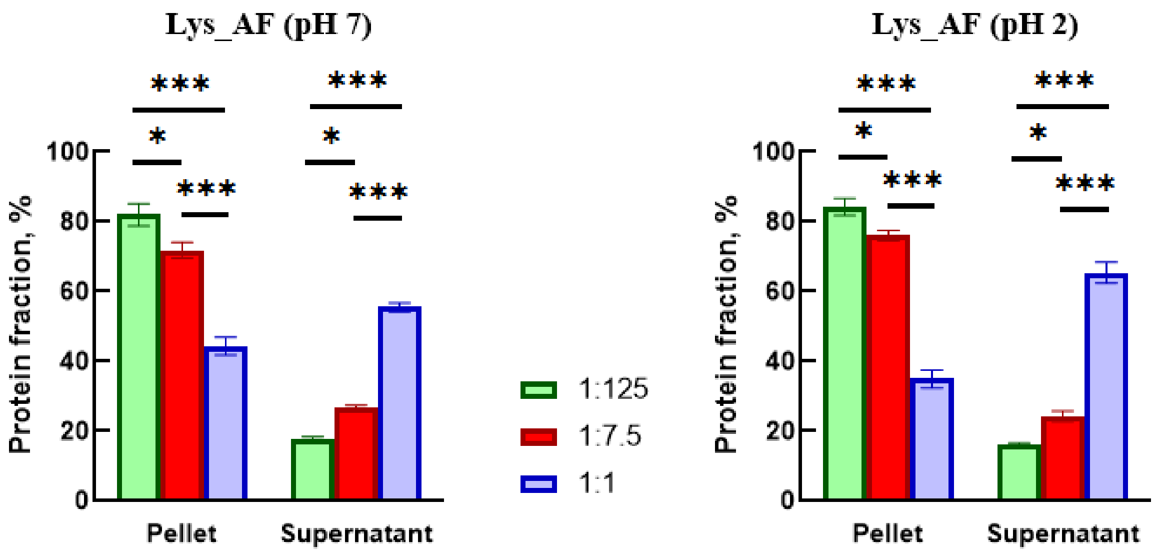

Figure S5. Redistribution of the protein between supernatant and pellet obtained by centrifugation of amyloid fibrils formed from lysozyme at pH 7 (Lys_AF (pH 7)) and at pH 2 (Lys_AF (pH 2)) treated with trypsin. Experiments are carried out at different ratio of trypsin to amyloid fibrils: 1 to 125, 1 to 7.5 and 1 to 1. Data are shown as average ± SEM. Statistical significance was estimated using one-way ANOVA with Tukey post hoc test. *P < 0.05, **P < 0.01, ***P < 0.001.

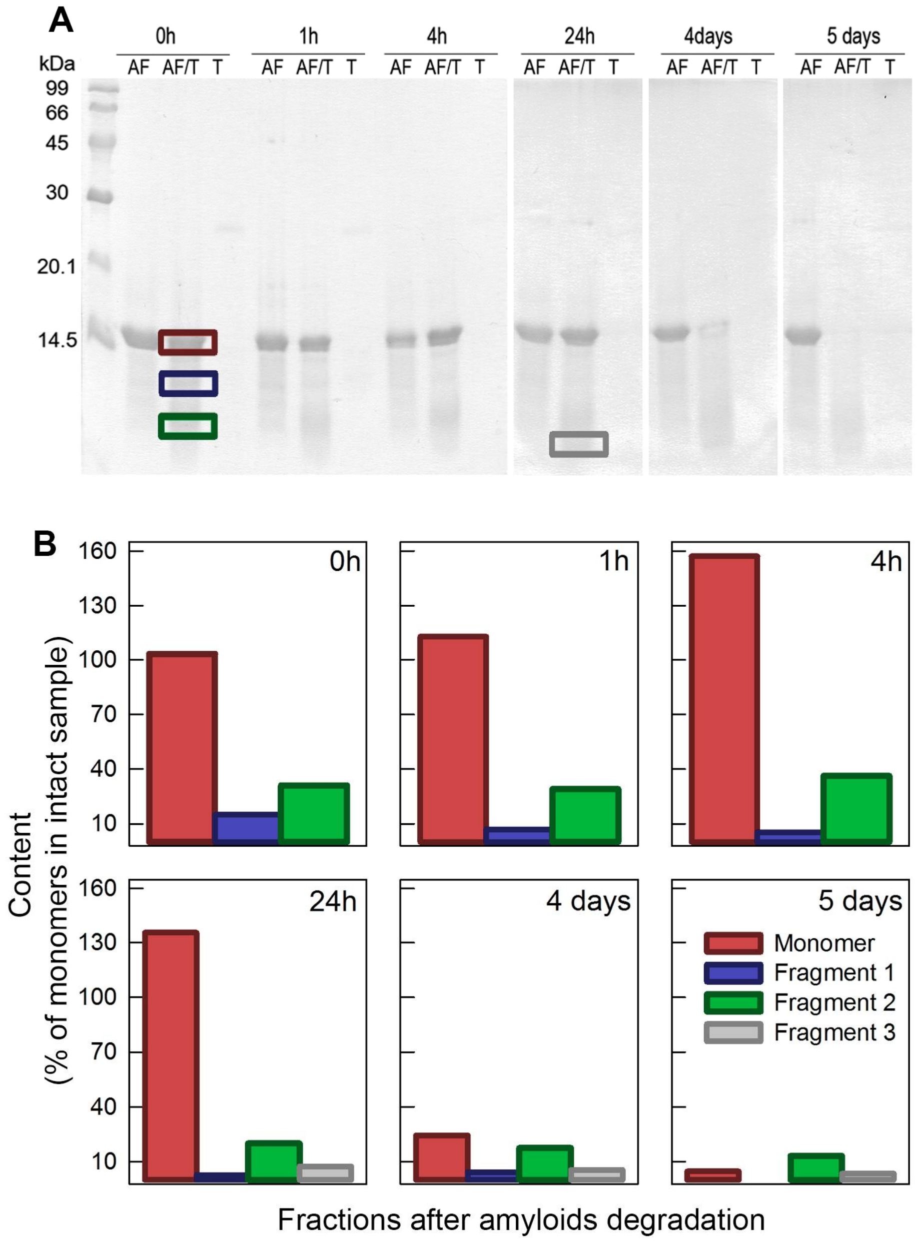

Figure S6. Trypsin induced degradation of lysozyme amyloid fibrils prepared at pH7. (A) SDS-PAGE under denaturing conditions of the amyloid fibrils alone (AF), after the addition of trypsin (AF/T) and trypsin alone (T). Solutions were incubated in PBS at 37 °C and aliquots were taken at different time points. Molecular weights of lysozyme and trypsin are 14.3 and 23.3 kDa, respectively. (B) Change in the content of lysozyme monomer and its fragments after amyloid fibrils degradation by trypsin evaluated in ImageJ. Data normalized to the content of monomer protein in the control sample with intact amyloid fibrils at the same time points. The position of lysozyme monomer and its three fragments on SDS-PAGE (Panel A) is indicated in frames the colors of which correspond to those in Panel B.

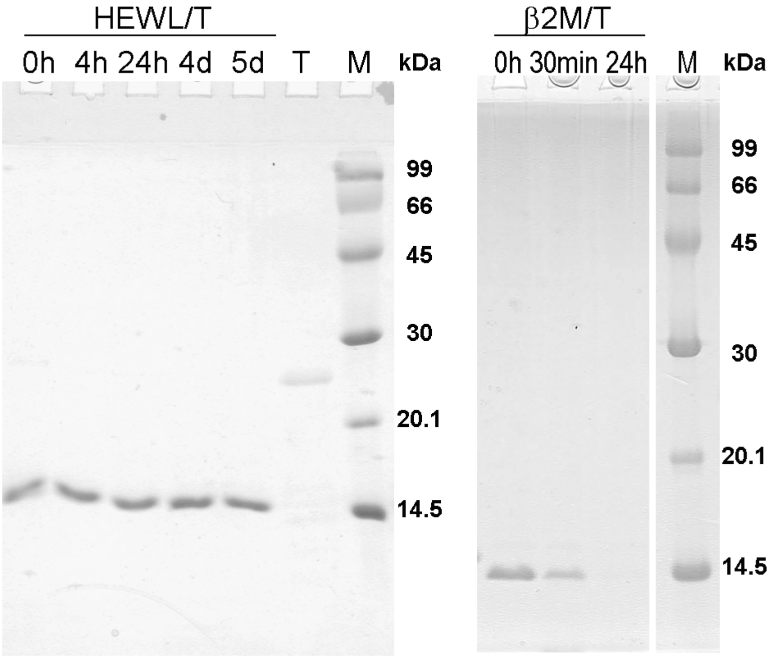

Figure S7. Trypsin digestion of lysozyme and beta-2-microglobulin (β2M) in their native state. Proteins at different time points after the addition of trypsin incubated in PBS at 37 °C were loaded on SDS-PAGE under denaturing conditions. Freshly prepared trypsin (T) at concentration 0.4 mg/ml was loaded as well. Molecular weight of lysozyme and beta-2-microglobulin is 14.3 and 11.8 kDa, respectively.

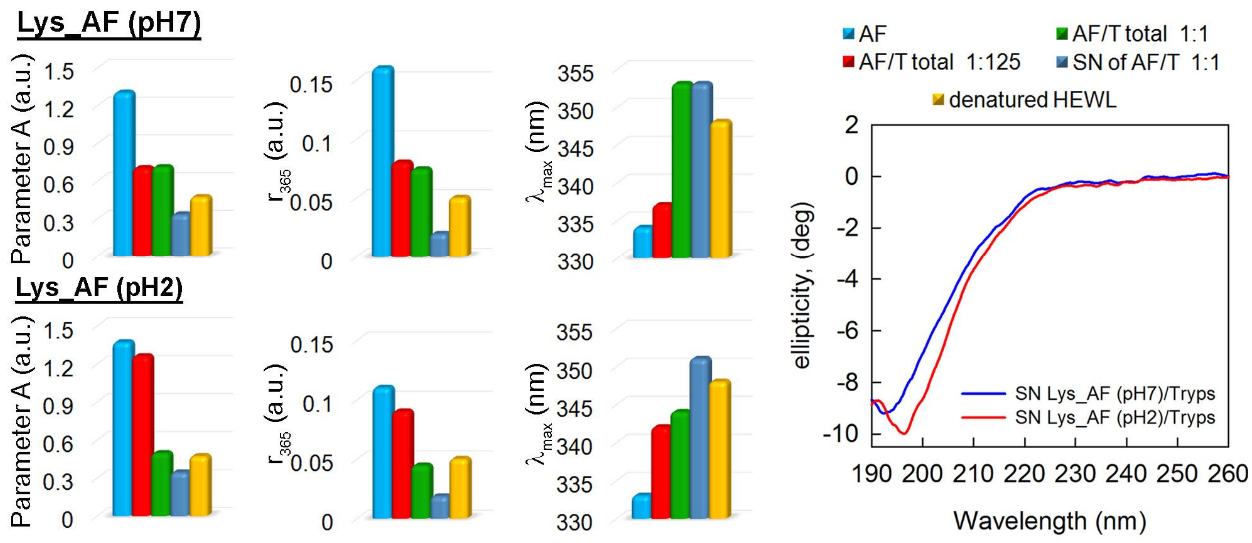

Figure S8. Spectral characteristics of amyloid fibrils formed from lysozyme at pH7 and at pH2 treated with trypsin. The Parameter *A*, fluorescence anisotropy (λ_{em} = 365 nm), wavelength of the fluorescence spectrum maximum (λ_{ex} = 295 nm) of mature fibrils (AF), amyloid fibrils treated with trypsin (AF/T) at ratio of 1:125 and 1:1, supernatant collected by centrifugation of amyloid fibrils treated with trypsin (SN of AF/T) at ratio of 1:1. The characteristics of denatured monomer lysozyme are shown for comparison. Additionally, the far-UV CD spectra for supernatant of amyloid fibrils treated with trypsin at ratio of 1:1 is represented.

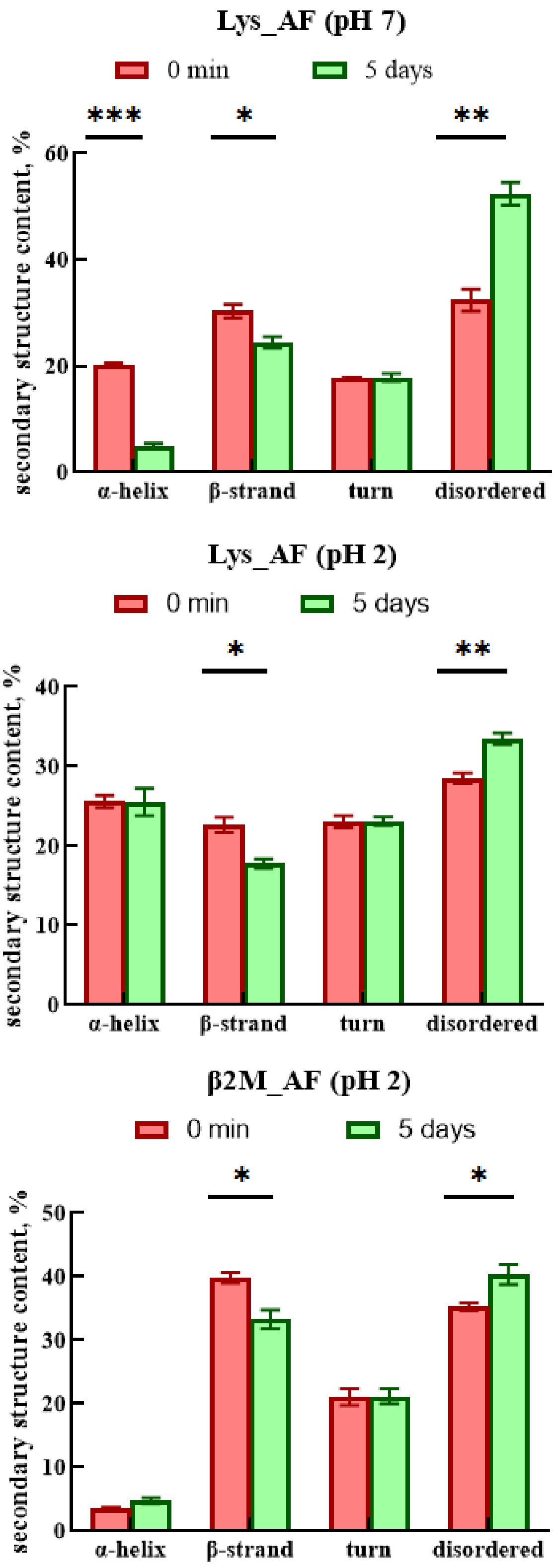

Figure S9. Trypsin induced changes of the secondary structure of amyloid fibrils formed from (A) lysozyme at pH7, (B) lysozyme at pH2 and (C) beta-2-microglobulin at pH2. Analysis was made on the basis of the far-UV circular dichroism (CD) spectra. Data are shown as average ± SEM. Statistical significance was estimated using Student t-test. *P < 0.05, ***P < 0.001, **P < 0.01.

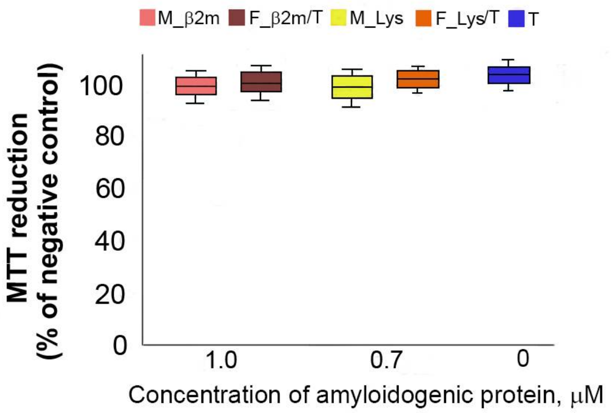

Figure S10. The effect of amyloidogenic proteins and their fragments on the cells determined by MTT assay. HeLa cells were exposed to monomeric beta-2-microglobulin (M_β2m) and lysozyme (M_Lys) and trypsin digested proteins fragments (F_ β2m/T and F_Lys/T), at indicated concentrations for 24 h. Data are expressed as medians and interquartile ranges, whiskers denote 1.5×IQR. Experiments were repeated at least 3 times with similar results.

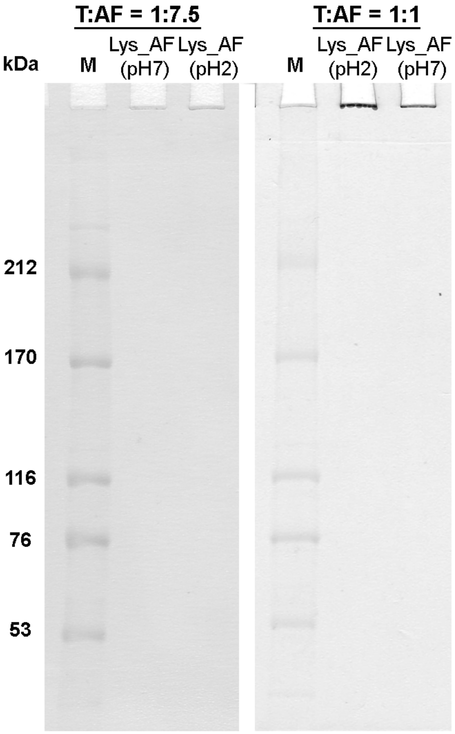

Figure S11. Identification of aggregates formed after treatment of amyloid fibrils with trypsin. The amyloid fibrils formed from lysozyme at pH7 (Lys_AF (pH7)) and at pH2 (Lys_AF (pH2)) after the adding of trypsin were incubated during 5 days and analyzed with pseudo-native SDS-PAGE in 8% polyacrylamide gel. Two different ratio of trypsin to AF was used: 1 to 7.5 (T:AF=1:7.5) and 1 to 1 (T:AF=1:1).

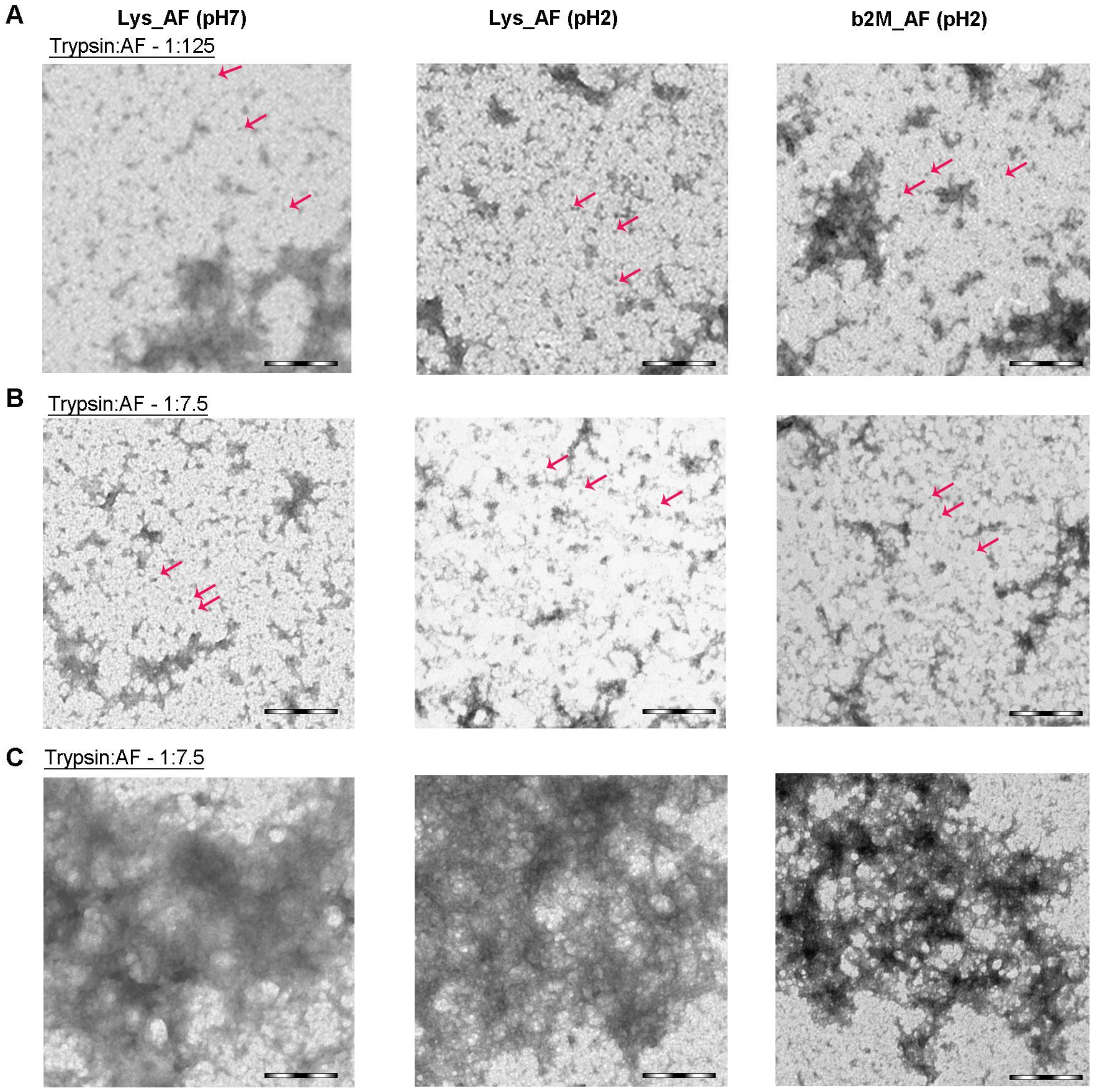

Figure S12. Characteristics of aggregates formed after treatment of amyloid fibrils with trypsin. The electron micrographs of supernatant collected by centrifugation of amyloid fibrils formed from lysozyme at pH7 and at pH2 and from beta-2-microglobulin at pH2. Scale bars are equal to 200 nm. Two different ratio of trypsin to AF was used: 1 to 125 (A) and 1 to 7.5 (B), (C). The estimated size of aggregates corresponds to 20 (shown by red arrows) or more monomeric protein molecules as evaluated by ImageJ. The large aggregates (C) were observed at both experimental conditions.

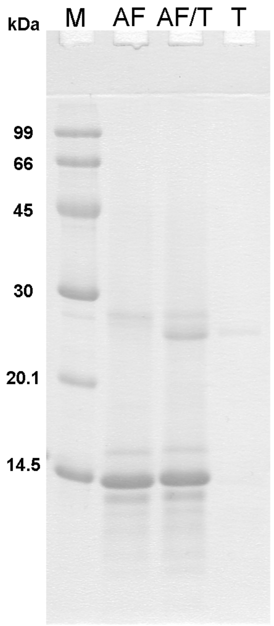

Figure S13. SDS-PAGE of beta-2-microglobulin amyloid fibrils mixed with trypsin. The amyloid fibrils alone (AF), after the addition of trypsin the proteolytic activity of which was quenched by PMSF (AF/T) and trypsin alone (T) were loaded into the wells. Molecular weights of beta-2-microglobulin and trypsin are 11.8 and 23.3 kDa, respectively.
